# GOTax: investigating biological processes and biochemical activities along the taxonomic tree

**DOI:** 10.1186/gb-2007-8-3-r33

**Published:** 2007-03-08

**Authors:** Andreas Schlicker, Jörg Rahnenführer, Mario Albrecht, Thomas Lengauer, Francisco S Domingues

**Affiliations:** 1 Department of Computational Biology and Applied Algorithmics, Max-Planck-Institute for Informatics, Stuhlsatzenhausweg, 66123 Saarbrücken, Germany

## Abstract

We describe GOTax, a comparative genomics platform that integrates protein annotation with protein family classification and taxonomy. User-defined sets of proteins, protein families, annotation terms or taxonomic groups can be selected and compared, allowing for the analysis of distribution of biological processes and molecular activities over different taxonomic groups. In particular, a measure of functional similarity is available for comparing proteins and protein families, establishing functional relationships independent of evolution.

## Abstract 2

GOTax, a novel web-based platform that integrates protein annotation with protein family classification and taxonomy, allows for an extensive assessment of functional similarity between proteins and for comparing and analyzing the distribution of protein families and protein functions over different taxonomic groups.

## Rationale

New opportunities for understanding biology at the molecular level have been created as a result of the complete sequencing and extensive annotation of different genomes. The scientific community is just starting to uncover the agents and mechanisms taking part in the molecular biology of different organisms as we identify their genes and gene products and the corresponding biological and molecular roles of these. The comparison of two different genomes allows for identifying the common and unique characteristics of each of the genomes and provides a way for transferring annotation from well characterized to less well characterized genomes. As more and more genomes from organisms of different species across the whole taxonomic tree are characterized, it becomes possible to compare not only two genomes, but also sets of genomes. Therefore, differences and similarities in the molecular biology between different taxonomic groups can be investigated in a systematic and objective way. For example, it becomes possible to identify the distribution of particular protein families or particular biological processes and molecular activities along the taxonomic or phylogenetic tree.

The comparison of different sets of genomes allows for identifying the processes, activities, and families unique to certain taxonomic groups or shared between taxonomic groups. A concrete application is the comparison between pathogenic and non-pathogenic bacteria, which provides insight into the mechanisms of pathogenicity. Another application is the comparison between human and different pathogens in order to identify features unique to the pathogens, a first step in the discovery of new drug targets. Comparative methods often rely on homology relationships, which are identified based on sequence similarity. More recently, new methods became available for comparing genes and gene products according to their functional annotation. In particular, measures of functional similarity of gene products have been proposed [1,2] that rely on the Gene Ontology (GO) [3] annotation. Several tools allowing such functional similarity searches have been developed [1,4,5].

We describe GOTax, a platform for investigating and comparing proteins, protein families, their activities and biological roles over the taxonomic tree. The platform includes GOTaxDB, a database integrating protein annotation [6], functional terms [3], protein family classification [7,8], and the taxonomic classification [9]. The database is accessed through the GOTaxExplorer tool, which provides a simple query language for querying the database and permits selecting arbitrary data sets. It also allows for the comparison of sets of proteins, protein families, and functional terms. In particular, it supports measures for the functional comparison of these sets. A functional similarity search tool (FSST) is also proposed, which provides a functional comparison of user-defined sets.

## The GOTax platform

**Figure 1:**
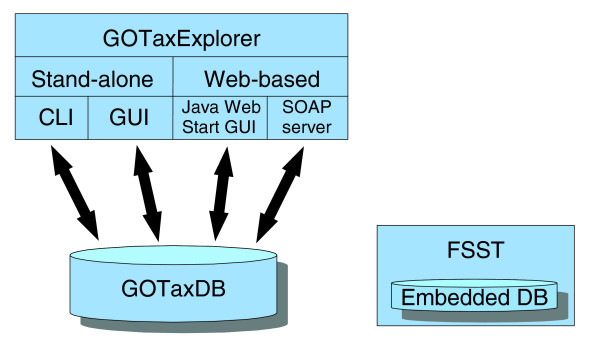
Schematic drawing of the GOTax platform. The schema shows the different components of the GOTax platform. GOTaxDB serves as central data storage that is queried by GOTaxExplorer. The stand-alone version of GOTaxExplorer comprises a GUI and a CLI. Additionally, GOTaxExplorer is available as a Java Web Start version. FSST is a module for calculating functional similarities between user-defined sets of proteins or protein families. It contains an embedded database making it independent from GOTaxDB.

The GOTax platform consists of four basic components (Figure 1). GOTaxDB integrates the different data sources and is queried either through the stand-alone version of GOTaxExplorer or through the Web Start version. The stand-alone version comprises a graphical user interface (GUI) and a command line interface (CLI). The forth component of GOTax is FSST, which consists of a query engine for functional similarity searches and an embedded database.

### GOTaxDB

We developed an intergrated database schema that was implemented using a MySQL [10] database server. It includes different data sources: UniProt, Pfam, SMART, GO, NCBI Taxonomy. They are all cross-linked, allowing the retrieval of entries from all sources based on SQL queries. The online version of GOTaxDB is updated every third month and new completely sequenced genomes will be added once a year. Since all cross-references between the data sources are provided by UniProt, the asynchronous release of the source databases does not affect updates of GOTaxDB. The database contains only data from completely sequenced species, currently about 260 species. The complete list of species is available on the worldwide web site [11]. In the following, single entries from any data source (proteins, Pfam families, SMART families, GO terms, taxa) are referred to as entities. A set of Java 1.5 [12] programs was implemented for importing data from downloaded flat files. The database containing the completed genomes is about 3 GB in size. Creating the database, including the computation of semantic similarity values for all GO terms, takes less than a week on an AMD Opteron 852 CPU with 2.5 Ghz. The semantic similarity data makes up two-thirds of the database, and is limited by the total number of GO terms and not by the number of genomes included in the database. Therefore, we expect the database to scale well while adding new completely sequenced genomes.

### GOTaxExplorer

GOTaxExplorer, the main tool for querying GOTaxDB, has been implemented in Java 1.5, providing a platform-independent software. The stand-alone version of GOTaxExplorer was successfully tested on Debian Linux, Red Hat Linux, Solaris 9, Windows 2000, and Windows XP. It comprises two user interfaces, a CLI and a GUI. The GUI is available either as a stand-alone application or as a Java Web Start application. Additionally, a SOAP [13] server is available. In order to take advantage of multi-processor systems, the semantic comparison of GO terms and the functional comparison of gene products are implemented multi-threaded.

#### GOTaxExplorer query capabilities

GOTaxExplorer supports four basic types of queries: selection of sets of entities; comparison of two sets of protein families; semantic comparison of two sets of GO terms; and functional comparison of two sets of proteins or protein families. Sets are selected by searching the different data sources integrated in the database and establishing relationships between the different data types (proteins, protein families, taxonomy, and GO) with a user-defined condition. This way the relationship between protein families and taxonomic groups can be investigated. A concrete example is determining the distribution of the PHP domain (PF02811) over the taxonomic tree. Moreover, GOTaxExplorer allows for finding the lowest common ancestor of species satisfying a query.

The relationship between protein families and functional annotation can also be investigated. An example is, "Which biological processes are annotated to proteins with a PHP domain?" Furthermore, queries relating proteins to a specific family and functional term are possible: "Select all proteins with the PHP domain that are involved in DNA replication." More complex queries can relate all integrated data sources to each other. This allows queries like "Which Pfam domains involved in DNA replication are present in bacteria and not in archaea?" The comparison of two sets of Pfam families shows which families occur in both sets or are unique to one of these sets. This also allows for a general functional comparison of these two sets according to the GO annotation of the Pfam families. This can serve as a guide for a more detailed functional comparison between the genes of two species. An example is the identification of the Pfam families associated with both yeast and human proteins.

The semantic comparison of two sets of GO terms uses a semantic similarity measure for finding similar and dissimilar terms in two sets of GO terms. The simple query "Which biological processes are present in *Saccharomyces cerevisiae *but not in human?" results in a list of GO terms. Let BP1 be a GO term mapped to a yeast protein and BP2 a GO term mapped to a human protein. Then the following cases can occur: BP1 equals BP2, BP1 is an ancestor of BP2, BP1 is a descendant of BP2, or BP1 and BP2 are both descendants of the same GO term. In the first case, the two terms are identical and, therefore, BP1 is not included in the result set. If BP1 is an ancestor of BP2, the true path rule implies that BP1 is also not included in the results. The true path rule states that a gene product annotated with one GO term can also be annotated with the term's ancestors in the graph. In the third case, the situation is not clear. In this case, the human gene product is annotated with the less detailed GO term, which can have several reasons. One possible explanation is that there is less knowledge about the human protein. However, it could also be the case that the fungal protein is a subclass of the more generic human protein. Alternatively, it is possible that there is more knowledge about the specific function of the human protein but it is not reflected by the annotation, or the exact functional term is still missing in GO. It remains unclear whether the human gene product is involved in the same process as the gene product from fungi or not. The fourth case presents a similar problem where the relationship between terms BP1 and BP2 is not clearly evident. In the latter two cases, the semantic similarity provides a basis for deciding whether the two terms are similar or not.

The functional comparison of two sets of proteins or protein families allows for identifying functionally similar proteins or protein families from different species. For this comparison, we use semantic similarity scores that have been previously described [2]. The *MFscore *and the *BPscore *assess the similarity of the molecular functions and biological processes, respectively, annotated to two proteins or protein families. The *funSim *score and the *rfunSim *score combine *MFscore *and *BPscore *into one measure. These scores allow for identifying the differences in the molecular biology of two closely related species, for example: "How functionally related are the proteins from *Saccharomyces cerevisiae *and *Schizosaccharomyces pombe*?" The functional comparison of protein families provides a way to compare species or groups of species whose proteins are not properly annotated to GO, and instead relies on the GO annotation of Pfam. Since GOTaxDB has a rather complicated database schema, the direct SQL queries to the database are quite complex. Therefore, we implemented a new simple query language in GOTaxExplorer. This query language was designed to be as easy to use and as flexible as possible. This greatly simplifies the use of the platform. More details about the query language can be found in Additional data file 1.

#### GOTaxExplorer command line interface

The GOTaxExplorer CLI provides a light-weight interface with all query capabilities, but without input aids and result visualization. In addition to the query language, this version provides the option of using SQL for directly querying GOTaxDB. All results are printed onto the screen in a tabular format. The major advantage of this interface is that it can be integrated into scripts that automatically generate queries and further process their results. A batch file with commands can be used to query GOTaxDB or, alternatively, the query commands are read from the standard input. Furthermore, it is possible to integrate the CLI into other programs that access GOTaxDB in order to take advantage of the simple query language.

#### GOTaxExplorer graphical user interface

**Figure 2:**
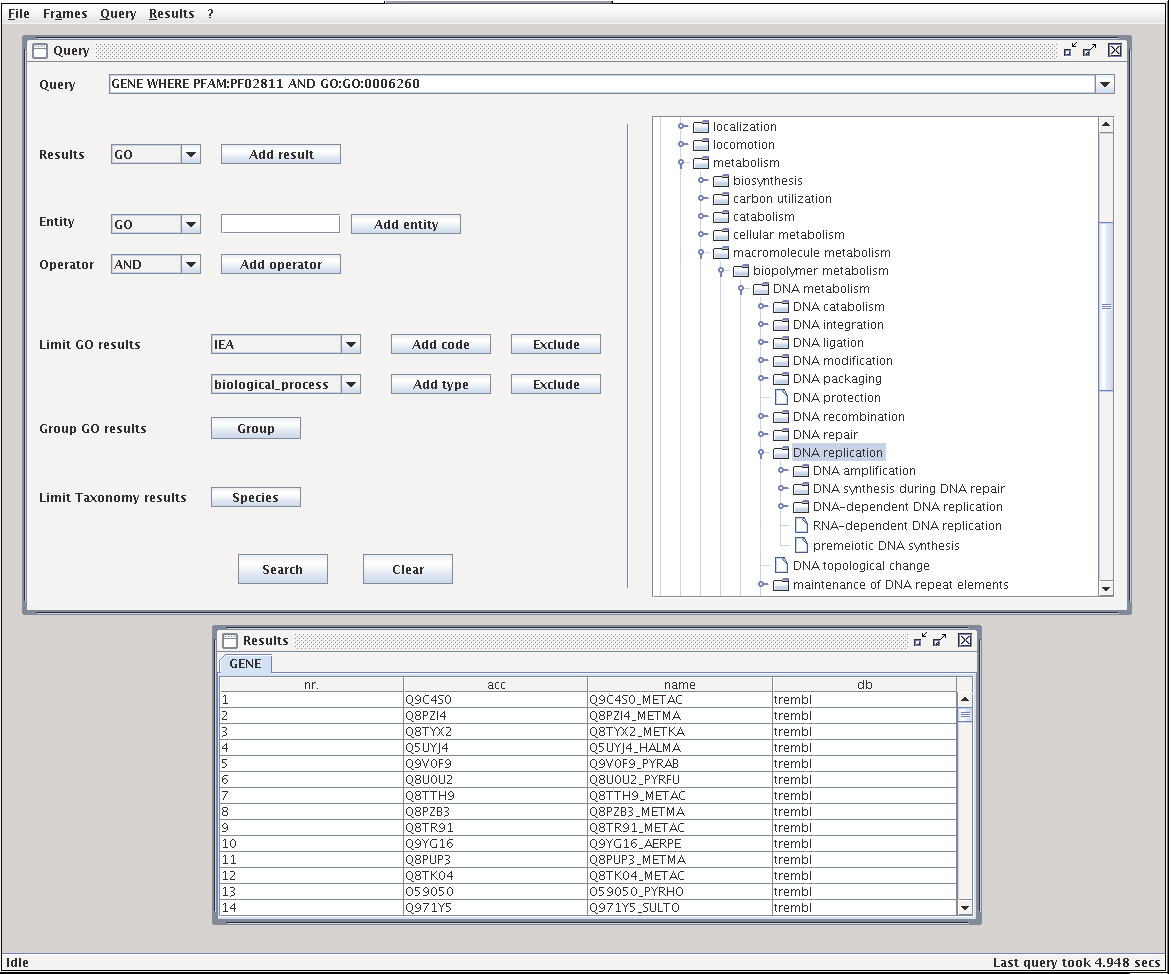
GOTaxExplorer's GUI. The screenshot shows the query interface with the results for the query "Which proteins with a PHP domain are annotated with DNA replication?" On the right side of the query interface is the graphical representation of the NCBI Taxonomy. The lower part shows the frame containing results from the current query.

The GUI provides a query interface that helps users to formulate queries without knowing the exact syntax of the query language (Figure 2). It also provides direct SQL access to GOTaxDB. The interface contains buttons for adding operators and entities to the query. Submitted queries are added to a query history for easy access. Furthermore, graphical tree representations of the GO ontologies and the taxonomic tree are available. These allow for browsing the hierarchies and facilitate finding a specific entry. Additional external information on an item can be accessed with a web browser. Since the tree representation permits only one parent for each node and the GO ontologies are directed acyclic graphs, all sub-graphs with more than one parent are replicated, unfolding the graph into a tree. Since the query language requires that entities are identified by their accession number in the source database, GOTaxExplorer provides the possibility of searching an entity by name in order to retrieve its accession number. Query results are presented in tables in the results frame. The tables can be sorted by different columns, and a popup menu provides access to external databases. Additionally, entities from previous results may be used in subsequent queries, facilitating further detailed investigation. Rows from result tables can be copied to the system clipboard, and complete result tables can be saved to standard text files. These files can easily be imported into databases or opened in spreadsheet applications.

**Figure 3:**
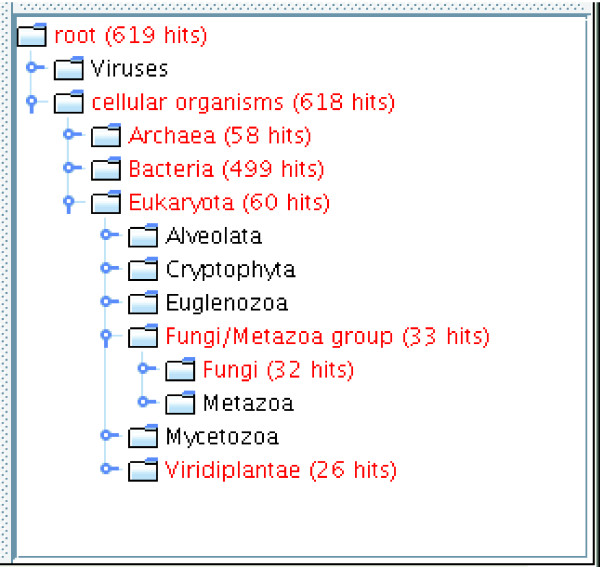
Screenshot of the taxonomic tree showing the distribution of the PHP domain (PF02811). The corresponding query is: TAX WHERE PFAM:PF02811.

**Figure 4:**
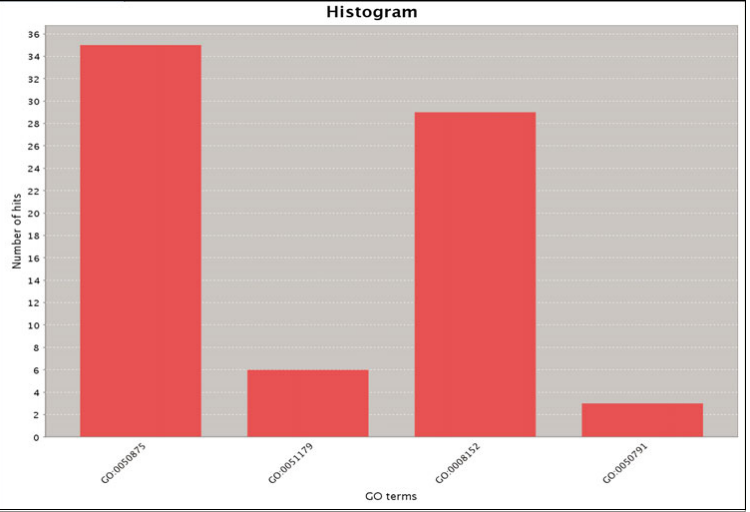
Screenshot of the histogram showing the grouping of the biological processes from yeast with a semantic similarity below 0.6 to any process from human. The groups correspond to 'cellular physiological process' (GO:0050875), 'localization' (GO:0051179), 'metabolism' (GO:0008152), and 'regulation of physiological process' (GO:0050791).

**Figure 5:**
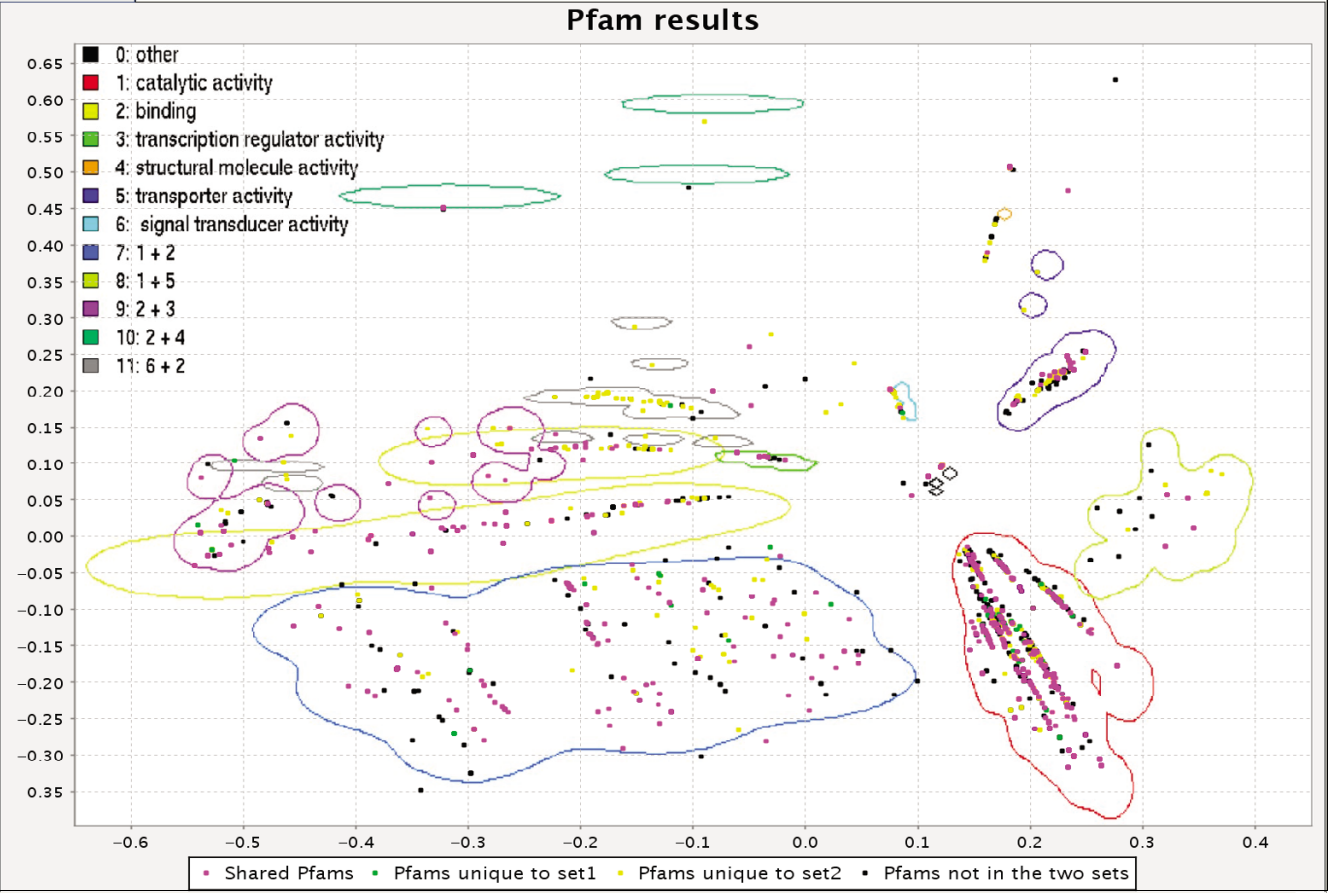
Screenshot of the map of the functional space of Pfam families showing the comparison of families between human and yeast. The map of the functional space was obtained by multidimensional scaling [2]. Pfam families shared between human and yeast are colored pink. Green dots indicate Pfam families unique to yeast and yellow dots represent families unique to human. Pfam families colored black do not occur in either human or yeast. The colored contour lines represent the regions of different functions. For more information on the map see [2]. The following query was used: PFAM WHERE TAX:4932 PFAMCP PFAM WHERE TAX:9606.

GOTaxExplorer provides the possibility to visualize taxonomy and GO results in the taxonomic tree or the GO tree (Figure 3). Additionally, a bar chart visualizes the grouping of GO results according to the given GO term list (Figure 4). Furthermore, the results from the selection of Pfam families or the comparison of two sets of Pfam families can be shown in a map of the functional space of all annotated Pfam families obtained by multidimensional scaling [2] (Figure 5). In this map, the Pfam families are grouped according to their functional similarity using the *MFscore*. The visualization of the results from a Pfam family query provides for quickly classifying found Pfam families into functional categories. In case of the comparison of two Pfam family sets, the families are colored according to the set they are contained in. This permits a functional classification of the families unique to one set, shared between both sets, and missing in both sets.

#### GOTaxExplorer Web Start version

GOTaxExplorer is also available as a Java Web Start client application [11]. Java Web Start is the most convenient way of using GOTaxExplorer because it does not depend upon a local database or application being installed by the user. It is started directly from a link on the GOTax web page and uses the most recent online version of GOTaxDB. The client application is stored and deployed on the user's computer and each time it is started Java Web Start checks for an update, which is automatically downloaded. This ensures that the user is employing the most recent release. The Web Start version provides the same functionality as the GUI of the stand-alone version with the exception of directly submitting SQL queries. Additionally, functional comparisons of proteins are not directly executed because they are computationally intensive. A request for these comparisons is sent to GOTaxDB. As soon as the results have been calculated, the user is informed via email.

#### GOTaxExplorer SOAP server

The SOAP server provides a standard interface for remote access to the online version of GOTaxDB. This interface allows for integrating the GOTax platform in other web services without the need for a locally installed version. The SOAP server provides the same query functionality as the Web Start version. We created a Web Services Description Language (WSDL) [14] file describing the capabilities of the SOAP server that can be used for implementing a compatible SOAP client. This file and additional documentation is available from the GOTax web site [11].

### Functional similarity search tool (FSST)

The FSST was implemented for comparing user defined sets of gene products or annotations. It supports all similarity measures and different output formats and its multi-threaded implementation takes advantage of symmetric multi-processing computers, decreasing runtime considerably. FSST is configurable using command line arguments and a configuration file. As input to FSST, the user can provide a database file in plain text format giving the reference entities and their GO annotation, and a query file with the same format defining the query entities with their annotation. It is possible to perform either an all-against-all or a one-to-one comparison of query entities against database entities. The results are written to a text file containing *BPscore*, *MFscore*, *funsim *score, and *rfunSim *score. Additionally, it is possible to obtain a similarity matrix for one of the scores from an all-against-all comparison. Different applications might require distances rather than similarities. Therefore, FSST is capable of transforming the scores into distances according to the formula

*dist*_*X*_(*A*,*B*) = 1 - *X*(*A*,*B*)   (1)

where *X *is either the *BPscore*, the *MFscore*, the *funsim *score, or the *rfunSim *score of the two gene products *A *and *B*. FSST is distributed with an embedded Apache Derby [15] database containing all semantic similarity values of GO terms. Therefore, FSST does not need GOTaxDB to be installed. The embedded version of Apache Derby has the advantage that it is completely administration free, and its deployment is completely hidden from the user.

## Introducing the rfunSim score

We developed a method for assessing the functional similarity of two gene products, the *funSim *score [2]. This score is based on the concept of the information content and uses the GO annotation of gene products [1,16,17]. The information content of a GO term is defined as the negative logarithm of the term's probability. This probability is computed as the relative frequency of a term in a large corpus of annotation. The probabilities for the GO terms were calculated based on the GO annotation in UniProt. Our semantic similarity measure for two GO terms (*sim*_*Rel*_) takes into account how close the two terms are to their lowest common ancestor as well as how detailed this lowest common ancestor is. It ranges from 0 for not related GO terms to 1 for highly similar terms. A *sim*_*Rel *_score below 0.5 indicates that the two GO terms are only distantly related [2]. The similarity between two gene products A and B with GO annotation *GO*^*A *^and *GO*^*B*^, respectively, is then calculated as follows. For each term in *GO*^*A*^, find the most similar term in set *GO*^*B*^, and calculate the average of their similarities as *rowScore*(*A*, *B*). Then, for each term in *GO*^*B *^find the term with the highest similarity from set *GO*^*A*^, and calculate the average as *columnScore*(*A*, *B*). The *GOscore*(*A*,*B*) is defined as:

*GOscore*(*A*,*B*) = *max*(*rowScore*(*A*,*B*), *columnScore*(*A*, *B*))   (2)

A *GOscore*(*A*,*B*) is calculated for BP (*BPscore*) and for MF (*MFscore*), respectively. Finally, the *funSim *score is calculated as:

$funSim=\frac{1}{2}\times [{\left(\frac{BPscore}{max(BPscore)}\right)}^{2}+{\left(\frac{MFscore}{max(MFscore)}\right)}^{2}],\;\left(3\right)$

where *max*(*BPscore*) and *max*(*MFscore*) denote the maximum possible score for biological process and molecular function, respectively. The *funSim *score ranges between 0 for completely unrelated gene products and 1 for gene products with identical functionality. Due to its definition, the *funSim *score is lower than the average of *BPscore *and *MFscore *in most cases. In order to obtain a more intuitive score, we define the *rfunSim *score for two gene products as

$rfunSim=\sqrt{funsim}=\sqrt{\frac{1}{2}\times [{\left(\frac{BPscore}{max(BPscore)}\right)}^{2}+{\left(\frac{MFscore}{max(MFscore)}\right)}^{2}].}\;\left(4\right)$

*rfunSim *ranges from 0 to 1 as *funSim*, but the values are up to 25% larger. Although the square root is a simple transformation, it changes the performance of the score. We tested how well the scores differentiate between protein pairs without sequence similarity and orthologous protein pairs. The ROCR [18] package for the statistical computing environment R [19] was used for calculating receiver operating characteristics (ROC) curves and the calibration error [20] for the classification task. The analysis shows that the *rfunSim *score achieves a better calibration error than the *funSim *score. The detailed analysis with examples of protein pairs can be found in Additional data file 1.

## Using GOTaxExplorer and FSST

In this section, we demonstrate the selection and comparison functionality of GOTax. First, the relationship between the PHP family domain and the other types of data is investigated. Comparisons are then performed at three levels with different examples: comparison of Pfam families, the semantic comparison of functional terms, and the functional comparison of proteins. For this analysis, the following databases where used: UniProt version 8.4, Pfam version 20, SMART extracted from InterPro [21] version 13, GO from August 2006, and the NCBI Taxonomy from 22 August 2006.

### Investigating the PHP domain

The PHP domain (PF02811) is a putative phosphoesterase domain and belongs to the Pfam clan 'Amidohydrolase superfamily'. This family includes bacterial DNA polymerase III proteins as well as histidinol phosphatases and uncharacterized proteins. The only member of this family with known three-dimensional structure is the hypothetical protein Ycdx from *Escherichia coli*. It has been shown that the active site of this protein contains three zinc ions [22]. The putative function of this domain is the hydrolysis of pyrophosphate during DNA synthesis.

First, we looked at the distribution of the domain over the taxonomic tree. Figure 3 shows the tree view of the taxonomy in GOTaxExplorer. As can be seen from this figure, this domain is widespread over all superkingdoms, archaea, bacteria and eukaryota. However, the domain does not occur in metazoa. Eukaryotic proteins with this domain belong to the class of phosphatases, and some are involved in DNA replication. We proceeded by identifying all biological processes in which proteins with the PHP domain participate. We found 11 different processes, which are listed in Additional data file 2. The results convey the idea that this domain is involved primarily in vital processes: 'DNA replication' (GO:0006260), 'DNA repair' (GO:0006281) and 'DNA recombination' (GO:0006310). We took a closer look at the category of 'DNA replication' (GO:0006260) and used GOTaxExplorer for obtaining the list of proteins that are annotated with this process and contain the PHP domain. The results are summarized in Additional data file 3. An example is the DNA-dependent DNA polymerase beta chain (Q99UW2) from *Staphylococcus aureus*. The list of results includes essential proteins from other pathogenic organisms, indicating that the PHP domain is a promising drug target.

### Comparison of Pfam families

We used GOTaxExplorer for the comparison of Pfam families found in human with Pfam families found in *S. cerevisiae*. The complete table with the results can be found in Additional data file 4. The search took less than 2 seconds, and the results show that human and yeast share 1,580 families, and that 1,478 Pfam families are unique to human and 252 are unique to yeast. The map of the functional space of Pfam families showing common families and families unique to one of the species is presented in Figure 5. As can be seen in this figure, many families annotated with 'binding' (GO:0005488) and 'signal transducer activity' (GO:0004871) are unique to human, for example, the small cytokines interleukin-8 like family (PF00048). Few protein families annotated with one of these categories are unique to yeast, such as the yeast mating factor alpha hormone domain (PF04648), or are common to both yeast and human, like the G-protein alpha subunit (PF00503) family.

Another example is the comparison of Pfam families found in proteins from human and different human viruses. GOTaxDB contains proteins and annotation from 18 human viruses, including the hepatitis C virus (HCV), HIV, and influenza viruses. The comparison shows that 24 Pfam families are shared between the viral and human proteins, 132 families are unique to viruses, and 3,170 families are unique to human. A map of the functional space with the comparison results is included in Additional data file 1 (as Figure S4). The FtsJ-like methyltransferase domain (PF01728) occurs in human and in viral proteins for example. This domain occurs at the amino terminus of flaviviral NS5 protein and is hypothesized to be involved in viral RNA capping [23]. All shared and unique Pfam families are listed in Additional data file 5.

It is also possible to compare the Pfam families of two different taxonomic groups. One example is the comparison of Pfam families found in proteins from *Viridiplantae *and fungi. Fungi and *Viridiplantae *share 1,898 Pfam families while 487 families are unique to fungi and 700 are unique to *Viridiplantae*. The results of this comparison can be found in Additional data file 6.

### Semantic comparison of functional terms

We performed a semantic comparison between biological processes associated with *S. cerevisiae *proteins and biological processes annotated to human. First, we identified 382 biological processes found in yeast and not in human. Then, a semantic comparison was performed between the processes unique to yeast and the processes found in human. We found 100 yeast processes with a *sim*_*Rel *_score above 0.9 to a human process, indicating that there is a very similar process in human for each of these yeast processes. The biological process with the lowest semantic similarity of 0.14 is 'plasmid partitioning' (GO:0030541). There are 38 processes in yeast with a semantic similarity below 0.6 to any process occurring in human. Such low similarity values suggest that the best matching process in human is only distantly related to the yeast process. These 38 processes were grouped into the more general GO categories 'cellular physiological process' (GO:0050875), 'localization' (GO:0051179), 'metabolism' (GO:0008152), and 'regulation of physiological process' (GO:0050791) (Figure 4). The complete list with the results of the semantic comparison can be found in Additional data file 7.

A similar comparison of all biological processes annotated to proteins from fungi with processes from *Viridiplantae *was performed. There are 219 processes from fungi with a *sim*_*Rel *_score above 0.9. Additionally, there are 24 processes with a *sim*_*Rel *_score below 0.5, indicating that there are no similar processes in human and, therefore, the respective processes are likely to be unique to fungi. The complete results can be found in Additional data file 8.

### Functional comparison of proteins

**Figure 6:**
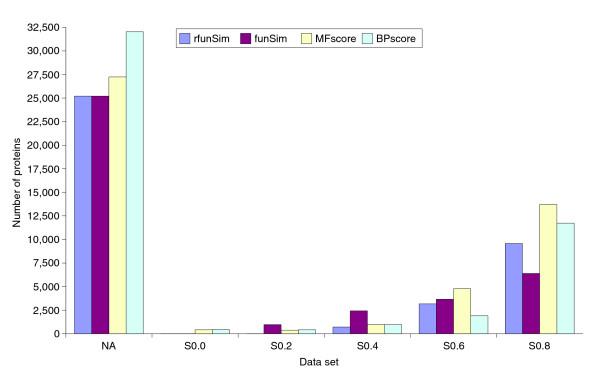
Functional comparison of *A. thaliana *proteins with *S. cerevisiae *proteins. Only the best hit (highest *rfunSim *score) for each yeast protein was taken into account for the score distributions. The following query could be used in GOTaxExplorer for obtaining these results: GENE WHERE TAX:3702 FUNSIM GENE WHERE TAX:4932.

We used FSST for a functional comparison of all proteins from *Arabidopsis thaliana *(NCBI Taxonomy id: 3702) and *S. cerevisiae *(NCBI Taxonomy id: 4932). UniProt contains 47,498 proteins from *A. thaliana*; out of these, 20,261 and 15,470 are annotated with MF and BP, respectively. From the 7,498 *S. cerevisiae *proteins in UniProt, 4,070 and 4,467 are annotated with MF and BP terms, respectively. The complete output can be found in the Additional data file 9. Figure 6 shows the distribution of scores for the best hits of *A. thaliana *proteins. The NA column contains proteins for which the corresponding score could not be computed because of a lack of molecular function or biological process annotation. More than half of *A. thaliana *proteins have either no molecular function or biological process annotation. Most of the annotated proteins have a high functional similarity to a *S. cerevisiae *protein. However, there are some proteins with a *rfunSim *score between 0.4 and 0.6, indicating only distant functional similarity. One such example is the cytokinin dehydrogenase 6 precursor (Q9LY71) from *A. thaliana*. It is annotated with the process 'stomatal complex morphogenesis' (GO:0010103) and the function 'cytokinin dehydrogenase activity' (GO:0019139). The most similar protein from yeast is the dihydrofolate reductase, which is annotated with the process 'folic acid and derivative metabolism' (GO:0006760) and with the functions 'dihydrofolate reductase activity' (GO:0004146) and 'protein binding' (GO:0005515). These two proteins have a *rfunSim *score of 0.47. Both proteins have oxidoreductase activity, which translates into a *MFscore *of 0.664. However, the processes they are part of are not related at all (*BPscore *= 0.0).

## Comparison to other tools

There are other databases and programs that allow for selecting and comparing sets of entities and for performing functional similarity comparisons. However, none of these tools offers the same degree of functionality as the GOTax platform. On the one hand, the Sequence Retrieval System at EBI [24], Entrez at NCBI [9], the Pfam database [7], the SMART database [8], and the PANTHER database [25] combine proteins, protein families, and the taxonomy and allow selection and comparisons of sets of database entries. However, they do not provide functional similarity comparisons. The integrated bio-data warehouse BioDW at Fudan University integrates protein, protein family and functional annotation databases [4] and allows basic semantic similarity searches. However, this semantic similarity search is restricted to one GO term and does not assess the overall functional similarity of two proteins. On the other hand, existing tools for performing functional comparisons between proteins do not provide an integrated database for queries and comparisons. GO Graph is a downloadable program for calculating semantic similarity between GO terms and functional similarity between proteins [1]. GO Graph allows for comparing user defined sets of proteins but does not offer protein and annotation databases for searching. DynGO is a downloadable application for performing semantic searches for gene products annotated with similar GO terms [26]. The software needs to be installed locally and also requires a local annotation database that has to be set up by the user. The Gene Functional Similarity Search Tool (GFSST) is an online resource that provides functional similarity searches within the human and mouse proteomes from UniProt [5]. This allows searching for functionally similar proteins, but only within a limited subset of known and annotated proteins. A comparison of the query capabilities of the different tools is provided in Table 1.

**Table 1:** Comparison of query capabilities

	Proteins	Pfam	SMART	GO	Taxonomy	Semantic similarity	Functional similarity
GOTaxExplorer	b,f,c	b,f,c	b,f,c	b,f,c	b,f,c	b,f,-	b,f,-
UniProt	b,f,c	b,f,c	b,f,c	b,f,c	b,f,c	-,-,-	-,-,-
Pfam	b,f,-	b,f,-	-,f,-	-,f,-	b,f,-	-,-,-	-,-,-
SMART	b,f,-	-,f,-	b,f,-	b,f,-	b,f,-	-,-,-	-,-,-
PANTHER*	b,f,-	-,-,-	-,-,-	b,f,c	b,f,c	-,-,-	-,-,-
InterPro	b,f,c	b,f,c	b,f,c	b,f,c	b,f,c	-,-,-	-,-,-
BioDW	b,f,-	-,-,-	-,-,-	b,f,-	-,-,-	b,f,-	-,-,-
GOGraph	-,-,-	-,-,-	-,-,-	-,-,-	-,-,-	b,-,-	b,-,-
DynGO	b,f,-	-,-,-	-,-,-	b,f,-	-,-,-	-,-,-	b,-,-
GFSST^†^	b,f,-	-,-,-	-,-,-	b,f,-	-,-,-	-,-,-	b,-,-

*PANTHER provides data for only seven different species. ^†^GFFST provides data for only human and mouse. The table shows which information is accessible via the corresponding databases or tools. The entries in each cell stand for (in this order): b, search by; f, search for; c, may be combined with other types. Hyphens "-" indicate a lack of the corresponding functionality.

## Conclusion

The GOTax platform integrates proteins, protein families, Gene Ontology, and taxonomy into one database. It provides several interfaces for the selection and comparison of different sets of entities, and for performing functional comparisons of proteins and protein families. The major limitation of our approach is the lack of complete annotation for many genomes and especially of the proteins unique to certain taxa. Although several completely sequenced genomes are annotated, this annotation is not complete and is still changing. Restricting GOTaxDB to completely sequenced genomes is one way of trying to prevent such problems, but the user should also take into account that more reliable results are obtained for the genomes that are extensively annotated. The extent to which the genomes are annotated is provided on the GOTax web site [11]. The usefulness of the GOTax platform will increase as more and more annotation becomes available. Further development of the GOTax platform may include enhancements to the user interfaces and the query language that simplify their use. One such example is the visualization of the GO as a directed acyclic graph instead of a tree. Moreover, adding other data sources, like non-coding RNA, to the database may expand the application scenarios of the GOTax platform.

## Additional data files

The following additional data are available with the online version of this paper. Additional data file 1 contains a description of the query language used by the GOTax platform and an analysis of the *rfunSim *score. Additional data file 2 lists all biological processes annotated to proteins with the PHP domain (PF02811). Additional data file 3 contains a list of proteins annotated with 'DNA replication' (GO:0006260) and containing the PHP domain (PF02811). Additional data file 4 lists the results from the comparison of Pfam domains from yeast and human. Additional data file 5 contains the results from the comparison of Pfam domains from viruses and human. Additional data file 6 contains the results of the comparison of Pfam domains from *Viridiplantae *and fungi. Additional data file 7 lists the results of the semantic comparison of biological processes from yeast and human. Additional data file 8 contains the results of the semantic comparison of biological processes from *Viridiplantae *and fungi. Additional data file 9 contains the complete FSST output for the comparison of proteins from *Arabidopsis thaliana *and *S. cerevisiae*.

## Supplementary Material

Additional data file 1 Figure S1: Description of the query language used by the GOTax platform and an analysis of the *rfunSim *score Click here for file

Additional data file 2 Table columns are, in the following order: numbering, GO term accession number, GO term name, GO term type. Click here for file

Additional data file 3 Table columns are, in the following order: numbering, UniProt accession number, UniProt name, database. Click here for file

Additional data file 4 Table columns are, in the following order: numbering, Pfam accession number, domain name. Set 1 corresponds to yeast Pfams and set 2 to human Pfams. Click here for file

Additional data file 5 Table columns are, in the following order: numbering, Pfam accession number, domain name. Set 1 corresponds to virus Pfams and set 2 to human Pfams. Click here for file

Additional data file 6 Table columns are, in the following order: numbering, Pfam accession number, domain name. Set 1 corresponds to Pfams from *Viridiplantae *and set 2 to fungal Pfams. Click here for file

Additional data file 7 Table columns are, in the following order: numbering, GO term accession number, GO term name, GO term type, *sim*_*Rel*_, matched GO term accession number, matched GO term name, semantic similarity according to Lin, matched GO term accession number, matched GO term name. Click here for file

Additional data file 8 Table columns are, in the following order: numbering, GO term accession number, GO term name, GO term type, *sim*_*Rel*_, matched GO term accession number, matched GO term name, semantic similarity according to Lin, matched GO term accession number, matched GO term name. Click here for file

Additional data file 9 Complete FSST output for the comparison of proteins from *Arabidopsis thaliana *(NCBI Taxonomy id: 3702) and *S. cerevisiae *(NCBI Taxonomy id: 4932) Click here for file
