# Supplementary material for: GOTax: investigating biological processes and biochemical activities along the taxonomic tree
Source: Genome Biol. 2007 Mar 8;8(3):R33. doi: 10.1186/gb-2007-8-3-r33 (PMC1868936; doi:10.1186/gb-2007-8-3-r33)
Supplement: Additional data file 1 — Figure S1: Description of the query language used by the GOTax platform and an analysis of the rfunSim score [file gb-2007-8-3-r33-S1.pdf]

Supplement
GOTax: Investigating biological processes and biochemical activities along the taxonomic tree

Andreas Schlicker*[1], Jörg Rahnenführer[1], Mario Albrecht[1], Thomas Lengauer[1], Francisco S Domingues[1]

[1]Department of Computational Biology and Applied Algorithmics, Max-Planck-Institute for Informatics, Stuhlsatzenhausweg 85, 66123 Saarbrücken, Germany

Email: Andreas Schlicker*- schlandi@mpi-inf.mpg.de; Jörg Rahnenführer - rahnenfj@mpi-inf.mpg.de; Mario Albrecht - mario.albrecht@mpi-inf.mpg.de; Thomas Lengauer - lengauer@mpi-inf.mpg.de; Francisco S. Domingues - doming@mpi-sb.mpg.de;

*Corresponding author

GOTax query language

Selection of sets

Proteins (keyword: GENE), Pfam families (keyword: PFAM), SMART families (keyword: SMART), GO terms (keyword: GO), and taxa (keyword: TAX) are referred to as entities in the following. Selecting sets of entities is done using a query of the form:

```
<result> WHERE <condition> RESTRICT <limit> GROUP <groups>.
```

This simple query allows for defining arbitrary sets of any information contained in the database. $\langle results\rangle$ is a comma separated list of the different results (GENE, PFAM, SMART, GO, TAX) of interest to the user. The $\langle condition\rangle$ can be composed of entities, the boolean operators AND, OR, NOT, and parenthesis "()". Taxonomic groups present a problem for query evaluation. Every species is mapped to a set of entities, either proteins, domains or GO terms. Since a taxonomic group is a set of species, a group evaluates to a set of sets of entities. Therefore, the set of entities of a taxonomic group can be defined as the union or the intersection of the species sets. We use the union of the sets of the different species as default. However, the user can select the intersection of the sets. An entity is defined by its domain, "GENE", "PFAM", "SMART", "GO", or "TAX", and the accession number from the source database separated by a colon ":", e.g. "GO:GO:0006260" or "PFAM:PF02811". The condition can contain any combination of different entities that are connected with boolean operators. The $\langle limit\rangle$ clause allows restricting the computation of GO results to annotations with certain evidence codes or GO terms from specified sub-ontologies. Every GO annotation is supplemented by an evidence code that refers to the method that was used to infer the annotation. An evidence code is preceded with "EC:", e.g. EC:IDA. In order to restrict to a sub-ontology, the prefix "TT:" is used followed by the sub-ontology, e.g. TT:molecular_function. If the specified evidence codes or sub-ontologies are to be ignored, the keywords "EXEC" or "EXTT" have to be added, respectively. For the species taxonomy, the user can restrict the results to species excluding all upper-level taxa by using the keyword "SPECIES". The GROUP operator is only applicable to GO results. If given, the results of the query will be categorized according to the GO terms given in $\langle groups\rangle$ and, for each term in $\langle groups\rangle$, the number of descendants in the result list is calculated. An example for the selection of sets is:

```
GO WHERE TAX:1770 AND NOT TAX:9606
  RESTRICT TT:biological_process,
```

which selects all GO terms from *M. paratuberculosis* that are not present in human, and limits the evaluation to biological processes.

Semantic comparison

A semantic comparison of two sets of GO terms is performed with a query like:

```
GO WHERE <condition> RESTRICT <limit> SIM
  GO WHERE <condition> RESTRICT <limit>.
```

Again $\langle condition \rangle$ and $\langle limit \rangle$ clauses may be defined completely freely, enabling the selection of arbitrary sets of GO terms for the comparison. An example for such a query is:

```
GO WHERE TAX:1770 AND NOT TAX:9606 SIM GO WHERE TAX:9606.
```

This query calculates semantic similarity scores between all GO annotations from *M. paratuberculosis* that are not present in human with all human GO annotations.

Functional comparison

The functional similarity between two sets of gene products is performed with a query like:

```
<result> WHERE <condition> FUNSIM <result> WHERE <condition>.
```

$\langle result \rangle$ may consist of either proteins, Pfam or SMART domains. The condition allows to arbitrarily select sets of these entities. Therefore, not only complete proteomes can be compared, but also proteins or domains involved in specific biological processes of molecular functions for example. The query

```
GENE WHERE TAX:1770 AND GO:GO:00006260 FUNSIM
  GENE WHERE TAX:9606 AND GO:GO:00006260
```

performs a functional comparison between proteins from *M. paratuberculosis* involved in DNA replication and human proteins involved in DNA replication.

Pfam set comparison

Two sets of Pfam domains are compared with the following query:

```
PFAM WHERE <condition> PFAMCP PFAM WHERE <condition>.
```

The result is a list of domains appearing in both sets, unique to either one of the sets and domains not present in the two sets.

A complete discussion of the query language is given on the GOTaxExplorer homepage [11].

Analyzing the *rfunSim* score

The *funSim* score is a measure of the functional similarity of two gene products [2]. It uses the gene product annotation with Gene Ontology terms and is based on the semantic similarity between two GO terms as used by Lord *et al.* [1]. The *funSim* score is defined as

$$funSim = \frac{1}{2} \cdot \left[\left(\frac{BPscore}{max(BPscore)} \right)^2 + \left(\frac{MFscore}{max(MFscore)} \right)^2 \right], \tag{1}$$

and ranges between 0, indicating no functional similarity, and 1 for exactly matching functions. Due to this definition, the *funSim* score of a pair of gene products is usually lower than the average of their *MFscore* and *BPscore*. Therefore, we define the $rfunSim$ score as square root of the *funSim* score,

$$rfunSim = \sqrt{funSim}. \tag{2}$$

This results in higher functional similarity values for most gene product pairs. In the following, some examples for protein pairs illustrate the difference between *funSim* and $rfunSim$ scores.

The stress response protein bis1 (O59793) from *S. pombe* is annotated with the function "protein heterodimerization activity" (GO:0046982) and the process "response to stress" (GO:0006950). The high pH protein 2 (P39734) from *S. cerevisiae* is involved in the same process but annotated with "protein binding" (GO:0005515) as function. The *funSim* score of these two proteins is 0.655 and the $rfunSim$ score is 0.809. Since they are involved in the same process and "protein heterodimerization activity" is a descendant of "protein binding" in the GO graph, the $rfunSim$ score seems to more accurately reflect the true functional similarity.

The glucan endo-1,3-alpha-glucosidase agn1 precursor (O13716) from *S. pombe* is involved in "cell septum edging catabolism" (GO:0030995) and shows the function "glucan endo-1,3-alpha-glucosidase activity" (GO:0051118). Protein EGT2 precursor (P42835) from *S. cerevisiae* has "cellulase activity" (GO:0008810) and is annotated with the process "cytokinesis" (GO:0000910). These two proteins have a *funSim* score of 0.364 and a $rfunSim$ score of 0.603. Looking at the GO graph, it becomes evident that "cytokinesis" is an ancestor of "cell septum edging catabolism" and that the functions of the two proteins are related through the common ancestor "hydrolase activity, hydrolyzing O-glycosyl compounds" (GO:0004553). The close relationship between the functions and the process annotated to the two proteins suggests that the $rfunSim$ score is more accurately capturing the true relationship.

Phosphatidylinositol-4-phosphate 5-kinase fab1 (O59722) from *S. pombe* has the function "1-phosphatidylinositol-3-phosphate 5-kinase activity" (GO:0000285) in the process of "endocytosis" (GO:0006897). The 1-phosphatidylinositol-3-phosphate 5-kinase FAB1 (P34756) from *S. cerevisiae* has the same function, but is annotated with three different processes, namely "phospholipid metabolism" (GO:0006644), "response to stress" (GO:0006950), and "vacuole organization and biogenesis" (GO:0007033). Considering that the two proteins perform the same function although involved in completely unrelated processes, the $rfunSim$ score of 0.711 seems more accurate than the *funSim* score of 0.505.

In order to obtain a more objective assessment of the performance of the $rfunSim$ score in comparison with the original *funSim* score, we used the sets of Inparanoid orthologs (IO) and of protein pairs without significant sequence similarity (NSS) from [2]. The *funSim* and the $rfunSim$ scores of all protein pairs in both sets have been computed and used for estimating prediction performance. The receiver operating characteristics (ROC) curves allow for assessing the performance of the scores in predicting true positives (protein pairs in IO) and true negatives (protein pairs in NSS). The ROC curves were calculated and visualized using the ROCR package [18] (Figure S1 and S2). The two curves are identical, the only difference being that the score cut-off at a given true positive and false positive rate is higher for the $rfunSim$ score.

The calibration error of a score measures how well the score coincides with the true class membership [20]. It is calculated as follows: first, all protein pairs are ordered according to their score. Then the pairs 1 - 100 are put into a bin and the percentage of true positives in this bin is calculated. Following, the mean prediction is calculated and the absolute frequency between observed true positive frequency and predicted positives gives the calibration error for this bin. This computation is repeated for protein pairs 2 - 101, 3 - 102 and so on. The final calibration error is then the mean of all binned calibration errors. Protein pairs

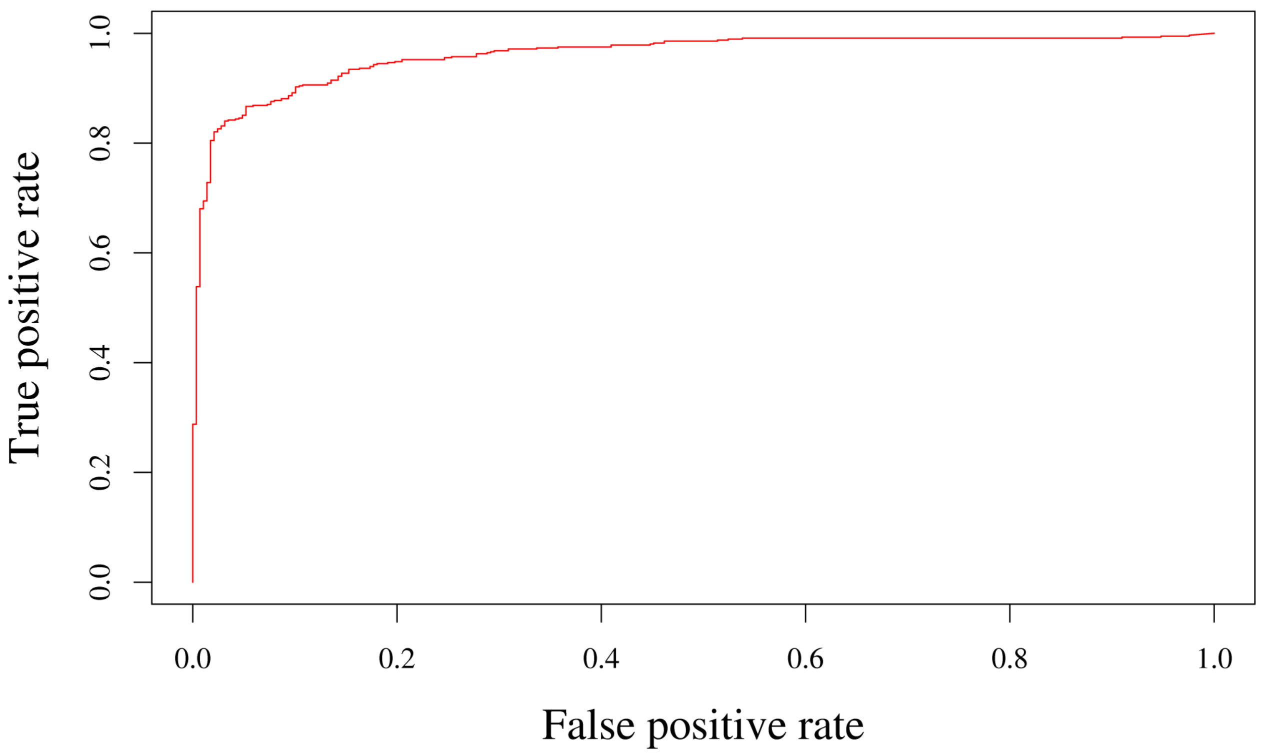

Figure S1: ROC curve for the *rfunSim* score. The curve shows the change in true positive rate with varying false positive rate.

with a score of 0.6 should belong to IO in 60% of the cases and to NSS in 40% of the cases for example and the calibration error measures the deviation of this ideal scenario. For this test, the *funSim* and *rfunSim* scores are interpreted as probability of two proteins to be functionally similar. ROCR was used for calculating and plotting the calibration error of both scores (Figure S2). The calibration error of the *rfunSim* score is smaller than the calibration error of the *funSim* score up to a score of approximately 0.75, and roughly equal thereafter. The results from the ROC curves and the calibration error analyzes support the intuition that the *rfunSim* score gives better results.

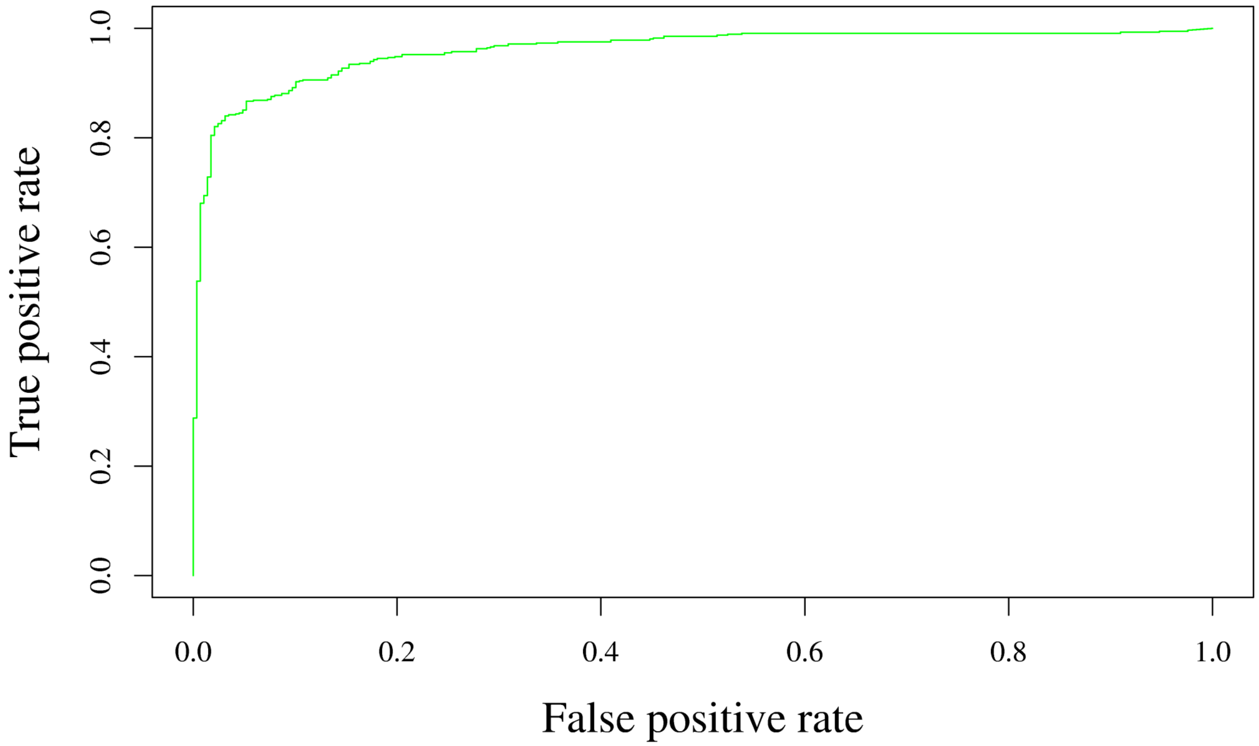

Figure S2: ROC curve for the *funSim* score. The curve shows the change in true positive rate with varying false positive rate.

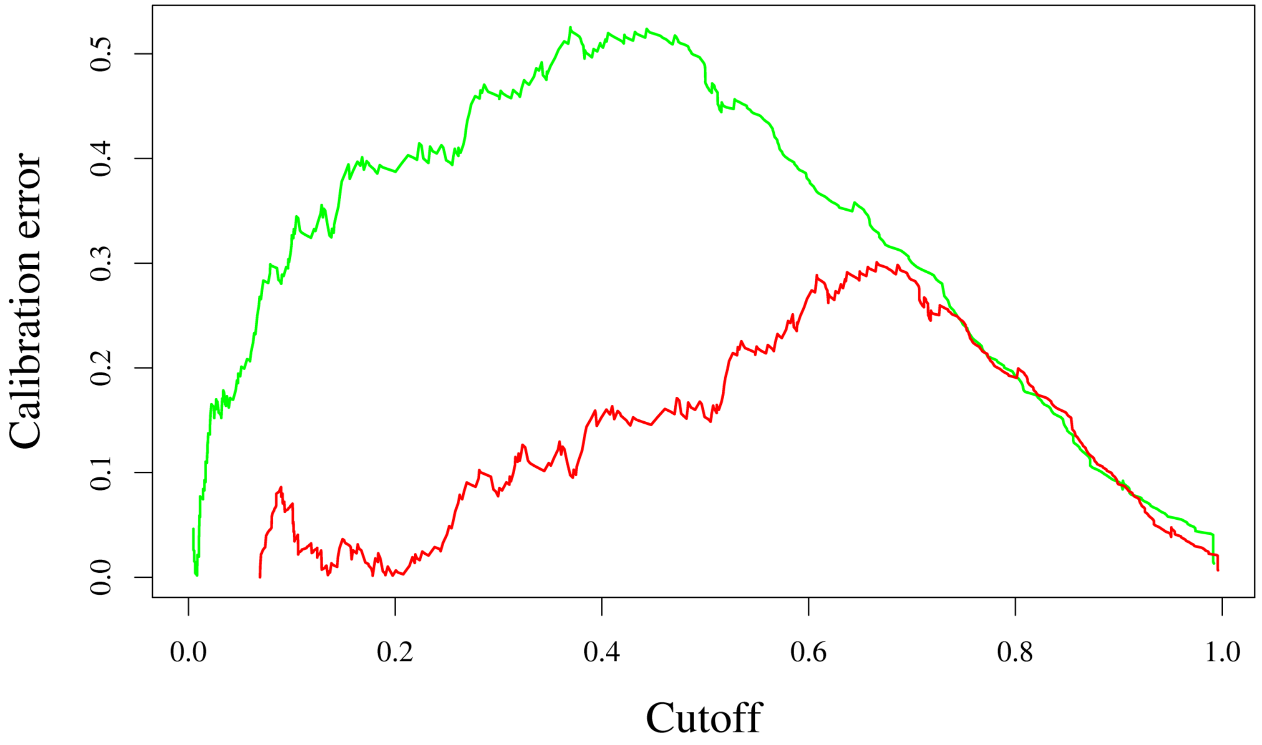

Figure S3: Calibration error for the *funSim* score and the *rfunSim* score. The green curve shows the error for the *funSim* score and the red line for the *rfunSim* score.

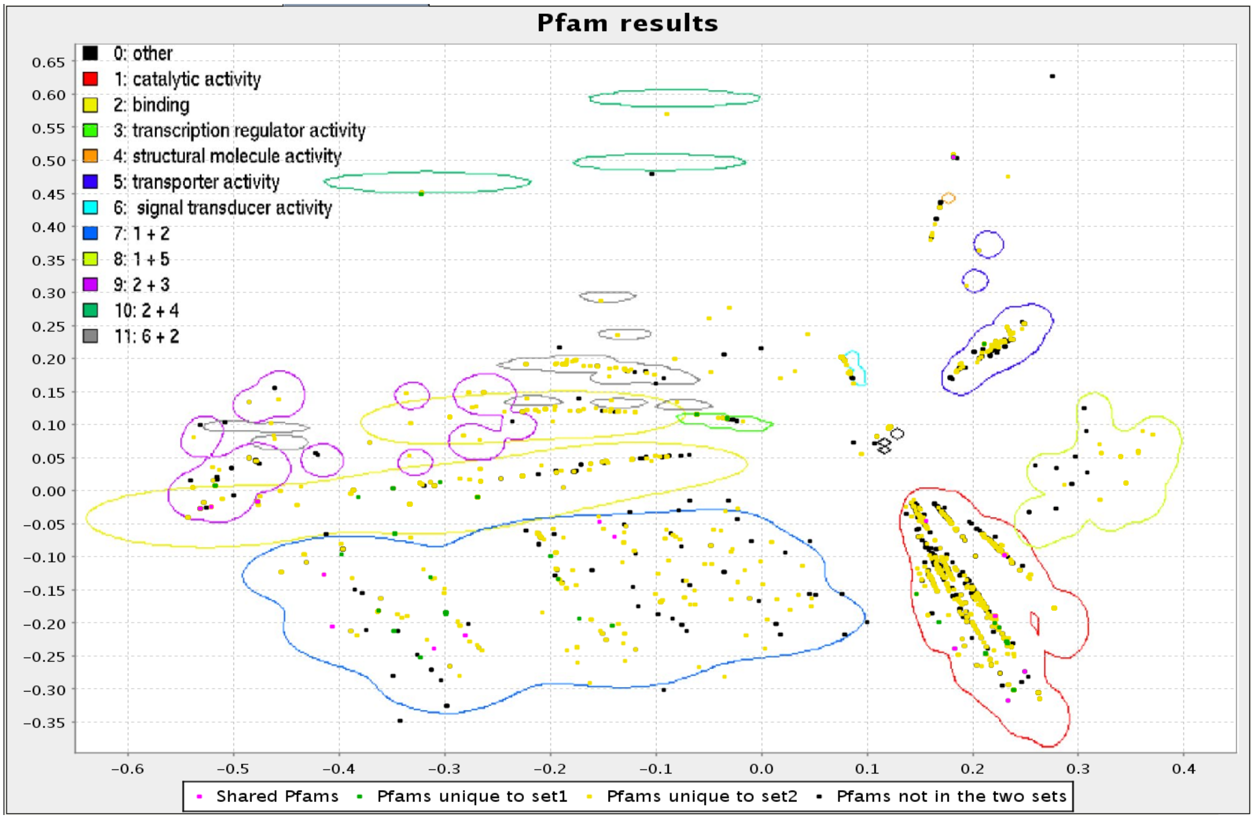

Figure S4: Map of the functional space of Pfam families showing the comparison of families between human and viruses. Pfam families shared between human and viruses are colored pink. Green dots indicate Pfam families unique to viruses and yellow dots represent families unique to human. Pfam families colored black do neither occur in human nor in yeast. The colored contour lines represent the regions of different functions. For more information on the map see [2].
